# Fluorometric Sensing and Detection of p-Nitroaniline by Mixed Metal (Zn, Ni) Tungstate Nanocomposite

**DOI:** 10.3390/nano13020362

**Published:** 2023-01-16

**Authors:** Fahad A. Alharthi, Hend Khalid Aldubeikl, Hamdah S. Alanazi, Wedyan Saud Al-Nafaei, Imran Hasan

**Affiliations:** Department of Chemistry, College of Science, King Saud University, Riyadh 11451, Saudi Arabia

**Keywords:** nitroaromatics, fluorescence, metal tungstate nanoparticles, sensors, quenching

## Abstract

Aromatic amines are important chemical intermediates that hold an irreplaceable significance for synthesizing many chemical products. However, they may react with substances excreted from human bodies to generate blood poisoning, skin eczema, and dermatitis disease and even induce cancer-causing high risks to human health and the environment. Metal tungstates have been proven to be highly efficient materials for developing various toxic gases or chemical detection sensor systems. However, the major factors of the sensors, such as sensitivity, selectivity, stability, response, and recovery times, still need to be optimized for practical technological applications. In this work, Ni-doped ZnWO_4_ mixed metal tungstate nanocomposite material was synthesized by the hydrothermal method and explored as a sensor for the fluorometric determination of p-nitroaniline (p-NA). Transmission electron microscopy (TEM) was used for the elucidation of the optimized particle diameter. Scanning electron microscopy (SEM) was employed to observe the surface morphological changes in the material during the solid-state reactions. The vibration modes of as-prepared samples were analyzed using Fourier-transform infrared spectroscopy (FTIR). The chemical bonding and oxidation states of individual elements involved in material synthesis were observed using X-ray photoelectron spectroscopy (XPS). The PL activities of the metal tungstate nanoparticles were investigated for the sensing of p-nitroaniline (p-NA). The obtained results demonstrated that ZnNiWO_4_ was more effective in sensing p-NA than the other precursors were by using the quenching effect. The material showed remarkably high sensitivity towards p-NA in a concentration range of 25–1000 μM, and the limit of detection (LOD) value was found to be 1.93 × 10^−8^ M for ZnWO_4_, 2.17 × 10^−8^ M for NiWO_4_, and 2.98 × 10^−8^ M for ZnNiWO_4_, respectively.

## 1. Introduction

Depending upon the demand of the developing society, chemical industries are producing nitroaromatic compounds in large scale; these are further utilized in the processing of pharmaceuticals, dyes, and pesticides [1,2]. Among the various nitroaromatic compounds, p-nitroaniline (p-NA) has been recognized as a key intermediate compound that is widely used in explosives, rubber, dyes, pesticides, and pharmaceutical products [3]. During chemical processing, p-NA can easily sneak into the environment as industrial waste and may contaminate the soil and surface water. Since p-NA has good solubility in water, it can be easily accumulated in humans as well as in aquatic animals and may produce toxic, mutagenic, and carcinogenic effects such as liver injury, skin eczema, diarrhea, methemoglobinemia, and anemia [4,5]. Thus, in view of its poor biodegradability and longtime, persistent nature, environmental protection agencies have categorized this as a priority pollutant. Therefore, there is a need for fast, robust, economical, and sensitive methods that can detect the minimum level of p-NA concentration in water. There are various methods reported in the literature for the selective and sensitive detection of various toxic nitroaromatic compounds such as UV–VIS spectrophotometry [6], electroanalytical [7], liquid chromatography [8], and fluorescent probes [9,10]. Out of these methods, the fluorescence detection method has attracted much attention due to its simple operation, rapid response, and high sensitivity as well as it being more cost effective and highly efficient [11,12].

Although, due to the presence of an electron-withdrawing group NO_2_, many of the nitroaromatic compounds are not intrinsically fluorescent [2]. Therefore, in this regard, fluorescence-based sensors have been proven to be very promising materials for the selective detection of nitroaromatic compounds even at trace levels [13]. One of the boons of nanotechnology is to provide advanced techniques to fabricate new materials that can detect, with enhanced efficiency and high sensitivity, nitroaromatic compounds [4,14]. The high sensitivity of these materials is attributed to the small size of the particles associated with nano dimensions and tailored morphology [15]. In recent times, metal tungstate-based nanomaterials have attracted the attention of researchers because of their robust application as scintillation detectors, photovoltaic electrochemical cells, humidity sensors, catalysts, and photoluminescent devices [16,17,18,19]. Zinc tungstate (ZnWO_4_) nanoparticles (NPs) have been recognized as one of the key important materials of the metal tungstate family because of high chemical stability, molecular and electronic versatility, and higher catalytic activity [20,21,22]. Owing to their crystallite structure (monoclinic wolframite) and low band gap (Eg = 3.2 eV), they have been utilized in various fields such as the magnetic, photo electrocatalytic, photocatalytic, and luminescent fields [22,23]. However fast they may be, the rate of electron hole recombination somehow restricts their photocatalytic as well as luminescent activity. To enhance this activity, a proper ion doping method was taken into consideration; this affected the band structure without mitigating the actual crystal structure [24,25,26]. The dopant in the material formed various doping levels, which could trap an electron hole pair, thus reducing the recombination rate and creating a new active site, which increased the redox activities of the nanomaterial [27]. Here, in the present study, Ni^2+^ was introduced in the crystal lattice of ZnWO_4_, which was attributable to small differences in their ionic radii such as Ni^2+^ (0.072 nm) and Zn^2+^ (0.074 nm) [28]. The resultant material, ZnNiWO_4_, was found to have small particle (16.68 nm) and monoclinic morphology, which reflected the high sensing efficiency as compared to the precursors, ZnWO_4_ and NiWO_4_.

In this work, the hydrothermal synthesis of ZnWO_4_, NiWO_4_, and ZnNiWO_4_ NPs was performed at 300 °C for 12 h. The nanoparticles were characterized by FTIR, XRD, SEM-EDS mapping, TEM-SAED, and XPS. The nanoparticles were explored for the fluorometric detection of nitroaromatic compounds from water. A comparative study among ZnWO_4_, NiWO_4_, and ZnNiWO_4_ was executed to determine whether to observe the effect of doping of Ni^2+^ ions on ZnWO_4_ fluorescence activity and crystal structure.

## 2. Methods and Material

### 2.1. Chemicals and Reagents

Sodium tungstate dehydrate (Na_2_WO_4_·2H_2_O, 98%) was purchased from Loba Chemie, Mumbai, India. Zinc nitrate hexahydrate (Ni (NO_3_)_2_·6H_2_O, 98%), nickel nitrate hexahydrate (Ni (NO_3_)_2_·6H₂O, 98%), and p-nitroaniline (p-NA, 99%) were purchased from Merck (Darmstadt, Germany). The ammonia solution (25%) was purchased from Otto Chemie (Mumbai, India). All the chemicals were used without further refinement, and distilled water was used for the preparation of the solutions.

### 2.2. Synthesis of Nanoparticles

#### 2.2.1. Synthesis of ZnWO_4_ Nanoparticles

The ZnWO_4_ nanoparticles were synthesized by using a standard hydrothermal method, reported elsewhere [22]. A total of 3 mmol each of sodium tungstate dihydrate and zinc nitrate hexahydrate were dissolved separately in 25 mL of distilled water and stirred for 15 min by a magnetic stirrer. After 15 min, 10 mL of 25% liquor ammonia was added dropwise in a way to maintain the pH of the mixture as 8–9. The mixture was transferred to a Teflon-lined steel autoclave and heated in a convection oven at 180 °C for 12 h at a heating rate of 5 °C/min. After the completion of the reaction, the as-synthesized ZnWO_4_ NPs were collected through centrifugation, washed several times with distilled water and absolute ethanol to remove any impurities, dried in a vacuum oven at 80 °C, and calcined at 600 °C for 4 h.

#### 2.2.2. Synthesis of NiWO_4_ Nanoparticles

The NiWO_4_ nanoparticles were synthesized by using a standard hydrothermal method, reported elsewhere [22]. A total of 3 mmol each of sodium tungstate dihydrate and nickel nitrate hexahydrate were dissolved separately in 25 mL of distilled water and stirred for 15 min by a magnetic stirrer. After 15 min, 10 mL of 25% liquor ammonia was added dropwise in a way to maintain the pH of the mixture as 8–9. The mixture was transferred to a Teflon-lined steel autoclave and heated in a convection oven at 180 °C for 12 h at a heating rate of 5 °C/min. After the completion of the reaction, the as-synthesized NiWO_4_ NPs were collected through centrifugation, washed several times with distilled water and absolute ethanol to remove any impurities, dried in a vacuum oven at 80 °C, and calcined at 600 °C for 4 h.

#### 2.2.3. Synthesis of ZnNiWO_4_ Nanocomposite

The mixed metal tungstate nanocrystals were synthesized by taking equimolar amounts (5 mmol) of each, Zn (NO_3_)_2_·6H_2_O, Ni (NO_3_)_2_·6H_2_O, and Na_2_WO_4_·2H_2_O, separately, dissolved in 25 mL of distilled water. The solutions were mixed and stirred by a magnetic stirrer for 15 min followed by the addition of 20 mL of 25% liquor ammonia to maintain the pH of the mixture up to 8–9. The mixture was transferred to a Teflon-lined steel autoclave and heated in a convection oven at 180 °C for 12 h at a heating rate of 5 °C/min. After the completion of the reaction, the as-synthesized ZnNiWO_4_ NPs were collected through centrifugation, washed several times with distilled water and absolute ethanol to remove any impurities, dried in vacuum oven at 80 °C, and calcined at 600 °C for 4 h.

### 2.3. Characterization of the Synthesized Materials

The M–O- and W–O-type bonds in the synthesized nanoparticles were evaluated by Fourier-transform infrared spectroscopy (FTIR) in the range of 4000–400 cm^−1^ by using a Perkin Elmer Spectrum 2 ATR (GOPRO Inc., San Mateo, CA, USA). The crystalline structure, crystallite size, and lattice phase of the synthesized nanoparticles were determined by using a Rigaku Ultima 1 V XRD diffractometer (Rigaku, Austin, TX, USA). The surface morphology of the material was studied using SEM integrated with EDX (SEM; JEOL GSM 6510LV, Tokyo, Japan) to obtain information about the elemental composition along with both the chemical composition and homogeneity of the synthesized ZnNiWO_4_ NPs. The particle size and their distributions were observed through a transmission electron microscope (TEM, TEM: JEM 2100, Tokyo, Japan). The chemical composition and elemental status of ZnNiWO_4_ NPs were evaluated by an X-ray photoelectron spectrophotometer (XPS, PHI 5000 Versa Probe III, Physical Electronics, Chanhassen, MN, USA). The fluorescence studies of the synthesized nanoparticles towards nitroaromatic compounds were observed through a fluorescence spectrometer, LS 55, PerkinElmer (Akron, OH, USA).

### 2.4. Sensing Experiment

Photoluminescence (PL) investigations of ZnWO_4_, NiWO_4_, and ZnNiWO_4_ NPs were performed at room temperature utilizing the Perkin Elmer LS55 fluorescence spectrophotometer. The as-synthesized 2 mg of nanoparticles were dispersed in 3 mL of methanol, and the photoluminescence spectra were recorded at various excitation wavelengths (360–410 nm) with a regular gap of 10 nm. The successive addition of a specific amount of p-NA was to evaluate the fluorometric detection capability of the as-synthesized nanoparticles. All the injected solutions were sonicated for 5 min before fluorometric detection. The λmax was observed at 390 nm before the analyte (p-nitroaniline) was added in the methanol suspension of as-synthesized nanoparticles. Then photoluminescence intensity of the of ZnWO_4_, NiWO_4_, and ZnNiWO_4_ NPs was recorded with the successive addition of a 25 μM solution of p-nitroaniline at a 320 nm excitation wavelength.

## 3. Results and Discussion

### 3.1. Material Characterization

**Figure 1:**
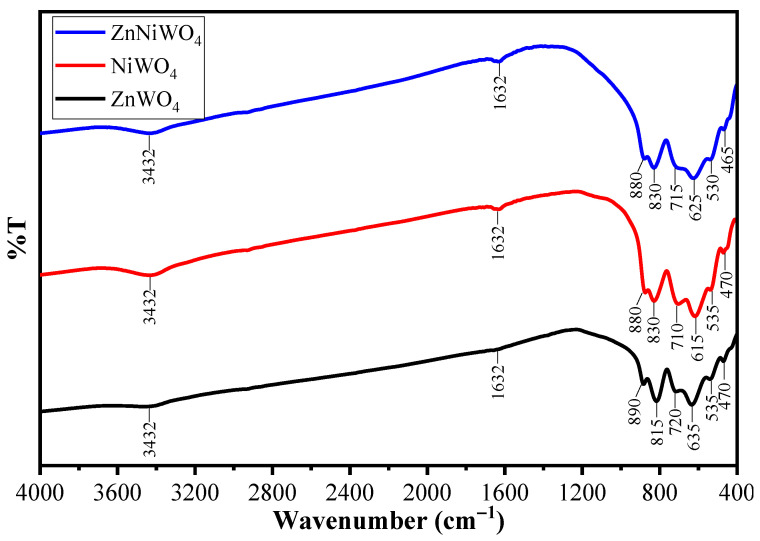
FTIR spectra of ZnWO_4_ (black line), NiWO_4_ (red line), and ZnNiWO_4_ (blue line).

Figure 1 shows the FTIR spectrum of the synthesized metals and the mixed metal tungstate (AWO_4_) nanoparticles measured in the range of 400–4000 cm^−1^. This spectrum was used to identify both the fingerprint and functional group regions in the sample. In the case of ZnWO_4_, 815–890 cm^−1^ belonged to the Zn–W–O vibrations, 720 cm^−1^ belonged to the stretching vibrations of the W–O bond, and 635 cm^−1^ belonged to the bending vibration of the W–O bond in WO_6_^6−^ octahedron, respectively [22]. The peaks at 470 and 535 cm^−1^ were assigned to uniform deformation modes of Zn–O and W–O bonds in ZnO_6_ and WO_6_ octahedrons, respectively [29]. In addition, the bands of the O–H stretch and H–O–H bending vibrations were located at 3432 and 1632 cm^−1^, which revealed that the synthesized samples contained a notable amount of some structural water and surface-adsorbed water [30,31]. In the fingerprint region, the absorption bands at 535 cm^−1^ corresponded to the NiO_6_ polyhedral in the crystal structure of NiWO_4_, 880 and 830 cm^−1^ were due to the vibration of the WO_2_ entity present in W_2_O_8_ group, and 710 and 615 cm^−1^ were due to the typical two oxygen bridge (W_2_O_8_)^−^ asymmetric stretching units [32]. The FTIR of the mixed metal tungstate ZnNiWO_4_ NPs represented all the peaks pertaining to ZnWO_4_ and NiWO_4_ considering 530 cm^−1^ as the Zn–O and 465 cm^−1^ as the Ni–O molecular vibrations [33].

**Figure 2:**
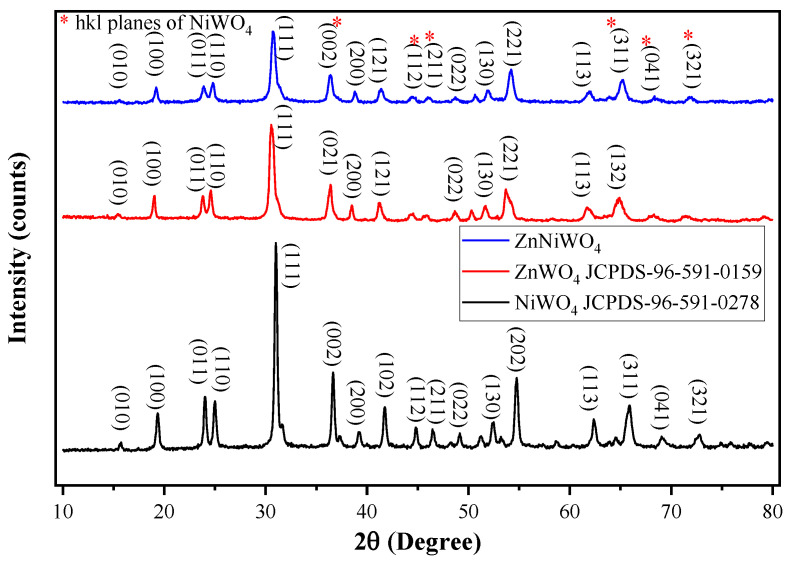
X-ray diffraction pattern of ZnWO_4_, NiWO_4_, and ZnNiWO_4_.

Figure 2 shows the XRD pattern of the ZnWO_4_, NiWO_4_, and ZnNiWO_4_ prepared by the hydrothermal method at 180 °C for 12 h. The XRD spectra of ZnWO_4_ showed characteristic peaks at 2θ value of 15.43°, 19.03°, 23.77°, 24.50°, 30.50°, 36.46°, 38.45°, 41.20°, 48.68°, 51.61°, 53.70°, 61.76°, and 64.90°, which belonged to the Miller Indices (010), (100), (011), (110), (111), (021), (200), (121), (022), (130), (221), (113), and (132), respectively (JCPDs card no. 96-210-1675). All the diffraction peaks were readily indexed to a pure wolframite-type monoclinic phase. Then, NiWO_4_ showed characteristic peaks at 2θ value of 15°, 19.30°, 23.95°, 24.99°, 30.96°, 36.65°, 39.21°, 41.76°, 44.89°, 46.52°, 49.16°, 52.47°, 54.74°, 62.41°, 65.92°, 69.10°, and 72.70°, which belonged to the Miller Indices (010), (100), (011), (110), (111), (002), (200), (102), (112), (211), (022), (130), (202), (113), (311), (041), and (321), respectively (JCPDS card no. 96-591-0278), which represented a standard monoclinic structure. Finally, the XRD pattern of the ZnNiWO_4_ NPs showed peaks ascribed to the ZnWO_4_ at 41.39° (121) and 54.17° (221) and peaks ascribed to the NiWO_4_ at 36.37° (022), 44.45° (112), 46.13° (211), 65.16° (311), 68.39° (041), and 71.90° (321), respectively, which suggested that Ni was successfully doped in the solid matrix of the ZnWO_4_. The structure resulted as monoclinic but with reduced peak intensity due to the superposition of Ni in the crystal structure [20,22]. Further information about the crystallite size and dislocation density and the Scherrer equation was taken into consideration [34].

(1) $$D=\frac{0.9\;\times \;\lambda }{\beta \;\times \;\mathrm{Cos}\theta }$$

(2) $$\mathrm{Dislocation}\;\mathrm{density}\;(\delta )=\frac{1}{D^{2}}$$

(3) $${\mathrm{Interlayer}\;\mathrm{spacing}\;(d}_{111})=\frac{n\lambda }{2\mathrm{Sin}\theta }$$

(4) $$\%\mathrm{Crystallinity}=\frac{\mathrm{Area}\;\mathrm{under}\;\mathrm{the}\;\mathrm{crystalline}\;\mathrm{peaks}}{\mathrm{Total}\;\mathrm{area}}\times 100$$

where D is the crystallite size, λ is the characteristic wavelength of the X-ray, β represents the angular width in radian at an intensity equal to half of its maximum of the peak, and θ is the diffraction angle. The average particle sizes of the ZnWO_4_, NiWO_4_, and ZnNiWO_4_ NPs were 14.43, 15.81, and 13.67 nm, respectively; they were calculated by using Equation (1) and are given in Table 1.

**Figure 3:**
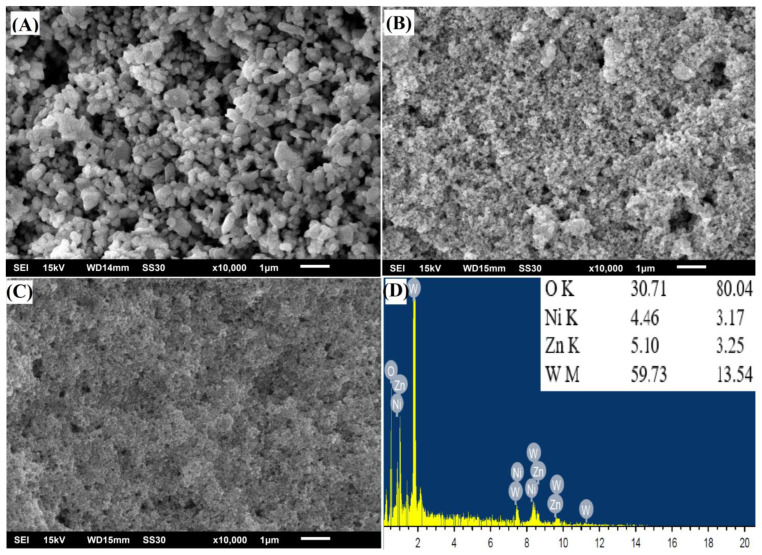
FESEM image of (**A**) ZnWO_4_, (**B**) NiWO_4_, (**C**) ZnNiWO_4_, and (**D**) EDX spectra of ZnNiWO_4_.

**Figure 4:**
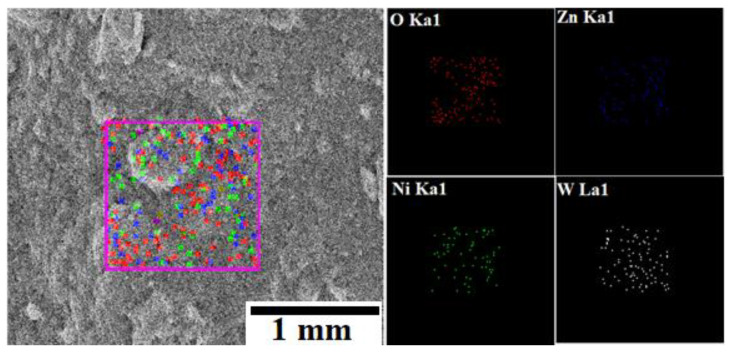
Selected area SEM image of ZnNiWO_4_ NPs showing the mapping of O, Zn, Ni, and W.

The morphology of the ZnWO_4_, NiWO_4_, and ZnNiWO_4_ NPs prepared by the hydrothermal method at 180 °C was evaluated by a scanning electron microscope (SEM). Figure 3A represents the SEM image of ZnWO_4_, in which particle are spherical in shape but executed at an agglomerated morphology. The SEM image of NiWO_4_ in Figure 3B represents a fluffy morphology with associated flakes in the particulates. The SEM image of the mixed metal tungstate ZnNiWO_4_ NPs in Figure 3C exhibited a collective array of agglomerated spherical-shaped particles with some fluffy appearances due to the mixing of Ni with ZnWO_4_. A semi-quantitative elemental analysis was performed on a selected area by an energy dispersive X-ray spectroscopy technique in an SEM chamber; this confirmed the presence of Zn (3.25%), Ni (3.17%), W (13.54%), and O (80.04%) elements, given in Figure 3D. Figure 4 shows the selected area mapping of the ZnNiWO_4_ NPs showing the uniform distribution of O, Zn, Ni, and W across the crystal structure.

**Figure 5:**
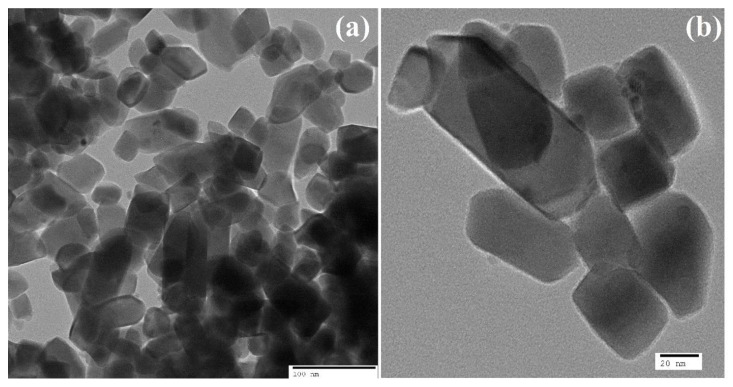

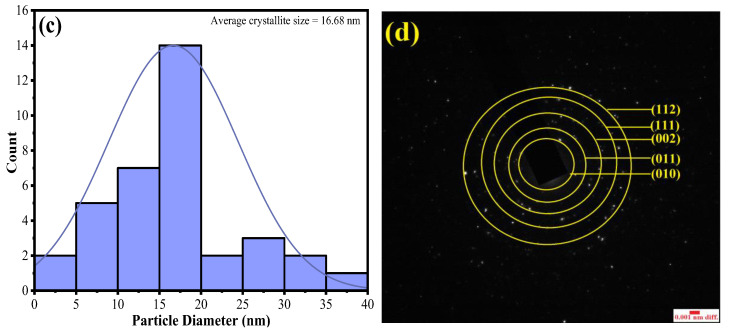
TEM images of ZnNiWO_4_ NPs at (**a**) 100 nm, (**b**) 20 nm magnification, (**c**) Gaussian distribution of the particle size, and (**d**) SAED of ZnNiWO_4_ NPs.

To evaluate the exact crystallite size and structure further, a transmission electron microscope (TEM) was used, and the results are given in Figure 5a,b. The TEM images of ZnNiWO_4_ represented an agglomerated monoclinic crystallites’ assembly with an average size of 16.68 nm (Figure 5c), which was also supported by the XRD results (13.67 nm). The SAED results, given in Figure 5d, also supported the Miller Indices values obtained for the XRD spectra of the ZnNiWO_4_ NPs.

**Figure 6:**
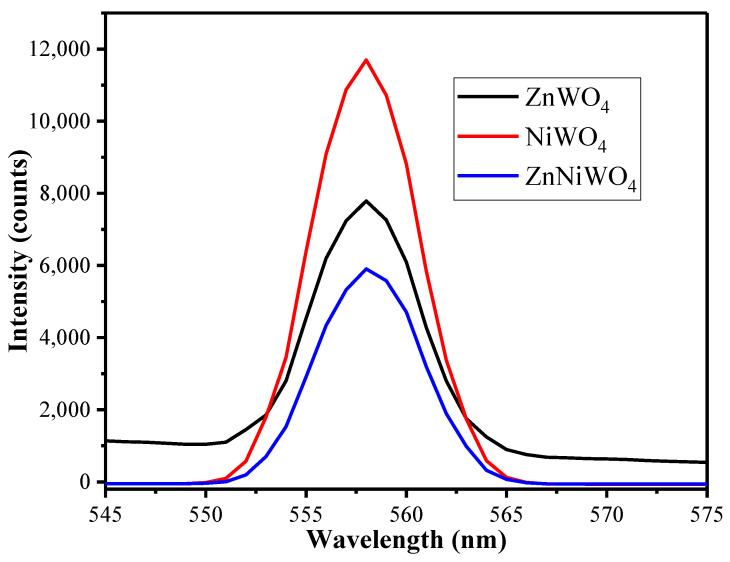
Photoluminescence (PL) spectra of ZnWO_4_, NiWO_4_, and ZnNiWO_4_ recorded at 320 nm excitation wavelength dispersed in methanol.

PL measurements are an effective method to monitor the process of photo-induced electron recombination and transfer. The PL of the ZnWO_4_, NiWO_4_, and ZnNiWO_4_ NPs was tested under 320 nm excitation, and the luminescence spectrum (Figure 6) spanned the range from 400 nm to 700 nm, showing a prominent emission peak at 510 nm. As can be seen in Figure 6, the PL intensity of the ZnNiWO_4_ NPs was lower than that of pure ZnWO_4_ and NiWO_4_. Since zinc tungstate has a wolframite monoclinic crystal structure, the luminescence properties of this crystal were noticeably different from NiWO_4_. The mixing of Ni with ZnWO_4_ resulted in emission associated with the radiative transitions between tungsten and oxygen within the (WO_6_)^6−^ molecular complex followed by a charge transfer from Ni^2+^ to Zn, which effectively suppressed the recombination of electron hole pairs [35].

**Figure 7:**
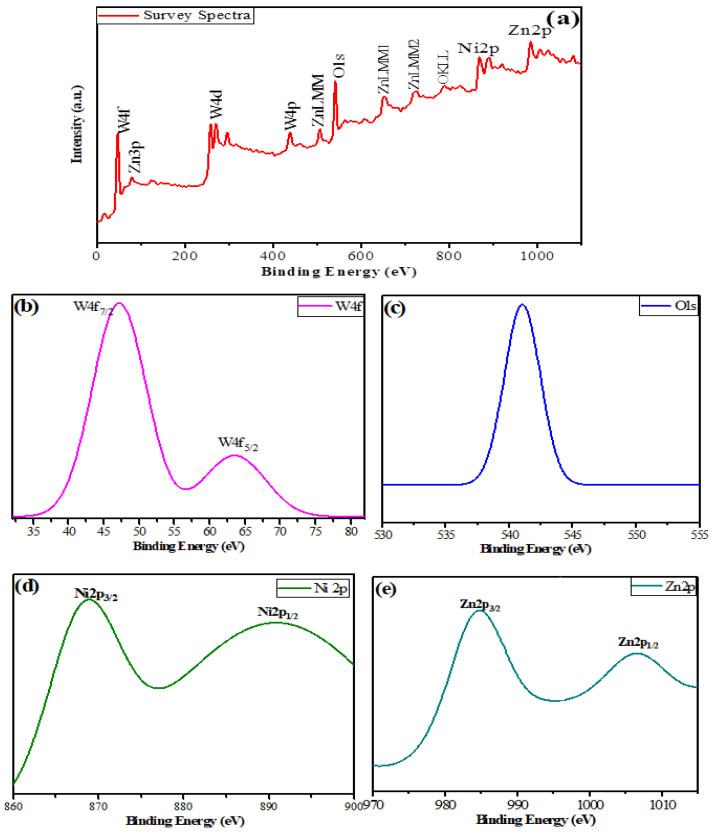
X-ray photoelectron spectroscopy (XPS) of ZnNiWO_4_ NPs’ (**a**) survey spectra, (**b**) W4f spectra, (**c**) O1s spectra, (**d**) Ni2p spectra, and (**e**) Zn2p spectra.

To study the chemical status and elemental composition of ZnNiWO_4_ NPs further, samples were investigated by X-ray photoelectron spectroscopy (XPS). The survey spectra, given in Figure 7a, revealed the elemental composition of NPs consisting of Zn, Ni, W, and O elements. To further evaluate the chemical status of elements in the crystal structure, high-resolution spectra for W 4f, O 1s, Zn 2p, and Ni 2p of ZnNiWO_4_ were also recorded (Figure 7b–e). Figure 7b consists of the W4f spectrum, which shows two spin-orbit doublets with peaks at 47.30 eV and 63.77 eV, representing the W 4f7/2 and W 4f5/2 belonging to the W^6+^ chemical state, respectively [36]. The O1s spectrum in Figure 7c resulted in a single broad peak at 541.03 eV, representing the oxygen coordination with Zn–O, W–O, and Ni–O, respectively [37]. Figure 7d displays the two major peaks at 868.95 eV and 890.71 eV, assigned to Ni 2p_3/2_ and Ni 2p_1/2_ spin-orbit peaks with their corresponding shake-up satellites. The Gaussian deconvolution of the Ni 2p_3/2_ line belonged to Ni^2+^ in the Ni (OH)_2_ peaks, while the Ni 2p1/2 line belonged to the Ni^2+^ ions, respectively [38]. The Zn 2p spectrum (Figure 7e) of ZnNiWO_4_ showed two peaks at 984.83 eV and 1006.40 eV, which were respectively attributed to Zn 2p1/2 and Zn 2p3/2 and suggested the presence of Zn^2+^ ions [39].

### 3.2. Photoluminescence Studies for Detection of p-Nitroaniline

#### 3.2.1. Effect of Solvent

**Figure 8:**
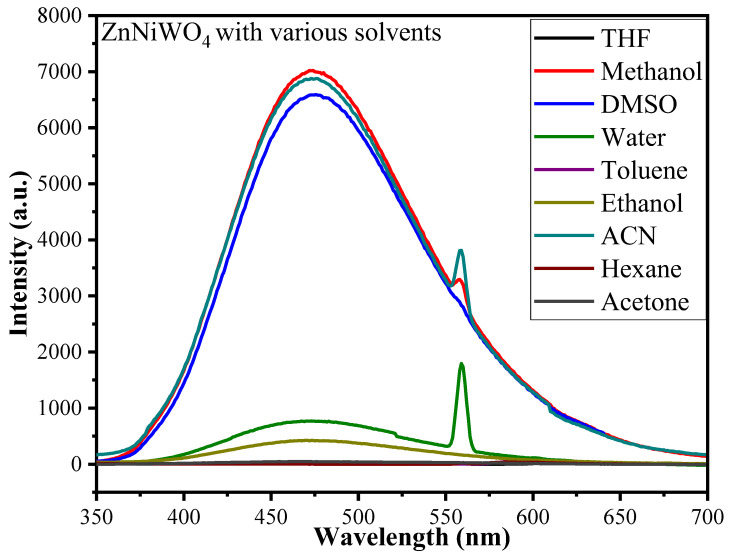
Solvent selection at which ZnNiWO_4_ exhibited maximum fluorescence intensity obtained at 320 nm excitation wavelength.

To evaluate the effect of a particulate solvent on the fluorescence intensity of the ZnNiWO_4_ NPs, experiments were conducted by immersing 2 mg of NPs in 5 mL of various solvents such as tetrahydrofuran (THF), methanol (MeOH), dimethyl sulfoxide (DMSO), deionized water (H_2_O), toluene (C_6_H_5_CH_3_), ethanol (EtOH), acetonitrile (ACN), hexane (C_6_H_14_), and acetone (CH_3_COCH_3_). The results suggested that, with different solvents, the emission peak intensity of the synthesized mixed NPs was found to be different. Figure 8 shows that the ZnNiWO_4_ NPs exhibited a maximum fluorescence emission intensity at 473 nm at an excitation wavelength of 320 nm with methanol (MeOH) followed by acetonitrile (ACN) and then DMSO. The high emission intensity belonged to radiative transitions between tungsten and oxygen within the (WO_6_)^6−^ molecular complex, which was influenced by the polarity of the solvent [39,40]. Therefore, based on the results, the ZnNiWO_4_ NPs with methanol were chosen as a blank for the detection of nitroaromatic compounds.

#### 3.2.2. Selectivity of Nitro-Compound

**Figure 9:**
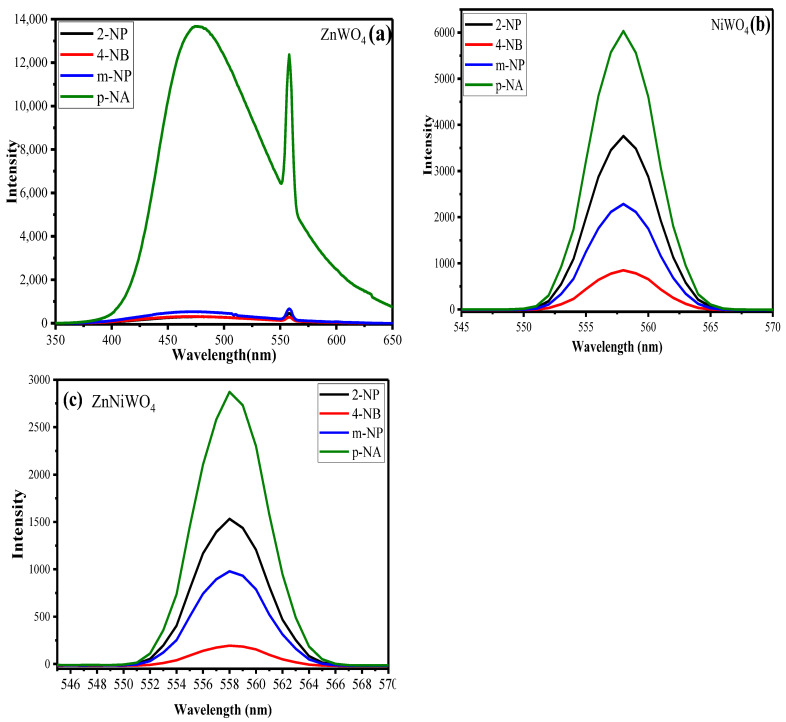
Effect of various nitroaromatics on the fluorescence spectra of (**a**) ZnWO_4_, (**b**) NiWO_4_, and (**c**) ZnNiWO_4_, recorded at 320 nm excitation wavelength.

The fluorescence-sensing properties of ZnWO_4_, NiWO_4_, and ZnNiWO_4_ for NACs, such as 2-nitrophenol (2-NP), 4-nitrobenzaldehyde (4-NB), m-nitrophenol (m-NP), and p-nitroaniline (p-NA), were investigated with methanol as a solvent medium. As shown in Figure 9a–c, strong emission peaks at 473 nm at 320 nm excitation wavelength were observed for p-NA by ZnWO_4_, NiWO_4_, and ZnNiWO_4_ in a methanol environment, which suggested that p-NA was most comprehended by metal tungstate and mixed metal tungstate nanoparticles. This phenomenon could have been caused by the hydrogen bond and the strong radiative transitions between tungsten and oxygen within the (WO_6_)^6−^ molecular complex [36,41]. The order of detection was found to be p-NA > 2-NP > m-NP > 4-NB. Therefore, p-NA was chosen for further experimental analysis.

#### 3.2.3. Effect of p-NA Concentration

**Figure 10:**
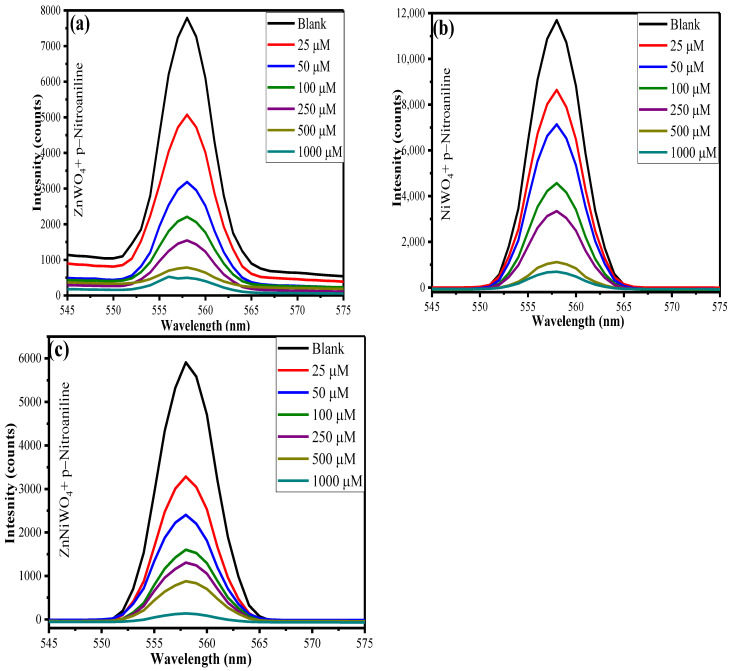
Effect of varying p-NA concentrations on fluorescence intensity of (**a**) ZnWO_4_, (**b**) NiWO_4_, and (**c**) ZnNiWO_4_, recorded at 320 nm excitation wavelength.

To explore the fluorometric detection ability of synthesized ZnWO_4_, NiWO_4_, and ZnNiWO_4_ NPs dispersed in methanol towards p-NA, fluorescence titrations were performed with an incremental concentration of p-NA from 25 μM to 1000 μM. It was seen (Figure 10a–c) that, with increase in concentration of p-NA, there was a slight decrease in the fluorescence intensity of the synthesized nanoparticles, suggesting an efficient quenching effect. The fluorescence quenching efficiency can be calculated by using [(F_0_ − F)/F_0_] × 100%, where F_0_ is the initial fluorescence intensity of dispersed nanoparticles in methanol and F is the fluorescence intensity in the presence of p-NA. The quenching effects for ZnWO_4_, NiWO_4_, and ZnNiWO_4_ NPs were found to be 93%, 94%, and 98%, respectively. The experiment showed that nitroaromatic compounds exhibited a stronger quenching effect, which was ascribed to the strong electron-withdrawing NO_2_ group [2,3,42]. Therefore, the mixed metal tungstate nanoparticles showed an improved quenching effect towards a higher concentration of p-NA as compared to its precursor.

**Figure 11:**
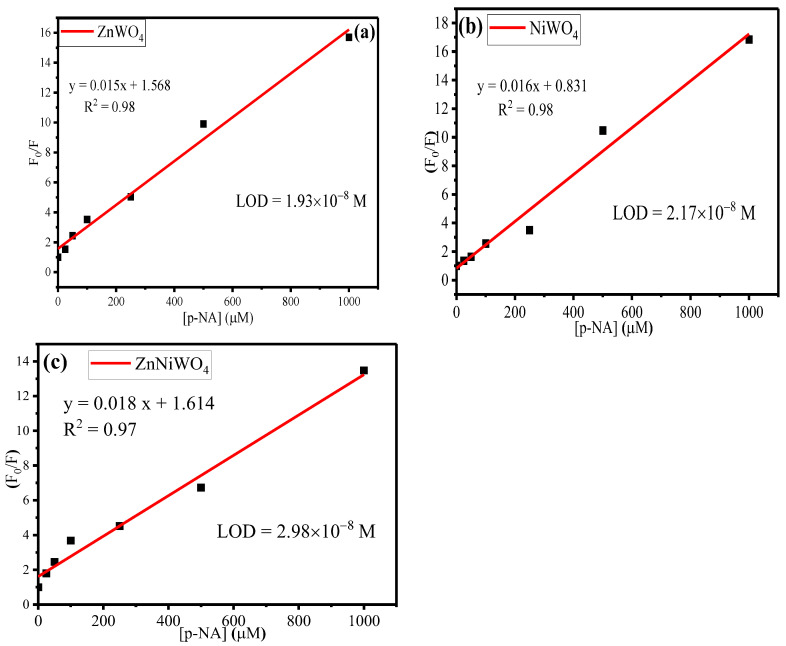
Stern–Volmer plots for varying p-NA concentrations in methanol for (**a**) ZnWO_4_, (**b**) NiWO_4_, and (**c**) ZnNiWO_4_ NPs.

The sensitivity of the sensor was evaluated by the Stern–Volmer equation, given by Equation (5) [43],

(5) $$\frac{F_{0}}{F}{=1+K}_{\mathrm{SV}}{\left[Q\right]}^{\;}$$

where Ksv is the Stern–Volmer constant, F_0_ and F are the fluorescence intensities before and after adding an analyte, respectively, and [Q] is the concentration of the p-NA. Figure 11a–c represents the Stern–Volmer plots for the ZnWO_4_, NiWO_4_, and ZnNiWO_4_ NPs. The high value of Ksv with a regression constant, given in Table 2, for ZnNiWO_4_ (0.018) as compared to ZnWO_4_ (0.015) and NiWO_4_ (0.016) suggested that the mixed metal tungstate nanoparticles were proven to be better sensors for p-NA. The limit of detection (LOD) value for p-NA was found to be 1.93 × 10^−8^ M for ZnWO_4_, 2.17 × 10^−8^ M for NiWO_4_, and 2.98 × 10^−8^ M for ZnNiWO_4_, respectively.

### 3.3. Anti-Interference Test

**Figure 12:**
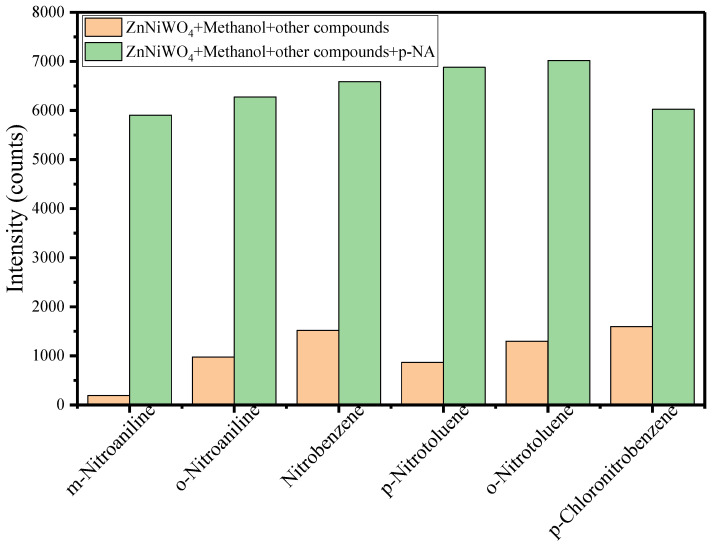
Anti-interference test for ZnNiWO_4_ for p-NA in presence of its analog.

The anti-interference capability of the synthesized nanocomposite sensor ZnNiWO_4_ was tested by comparing the PL intensities with p-NA and its analogs such as m-nitroaniline, o-nitroaniline, nitrobenzene, p-nitrotoluene, o-nitrotoluene, and p-chloronitrobenzene. The obtained results are given in Figure 12, in which the first column represents the PL intensity of the interfering agent (25 μM) with ZnNiWO_4_ and the second column represents the PL intensity of the interfering agent (25 μM), p-NA (50 μM) with ZnNiWO_4_ in a methanol environment. It can be seen from the results that the presence of interfering agents had no effect on the sensing capability of ZnNiWO_4_ towards p-NA, which suggested that the synthesized nanomaterial had a very good selectivity and sensitivity for p-NA.

### 3.4. Recyclability Test

**Figure 13:**
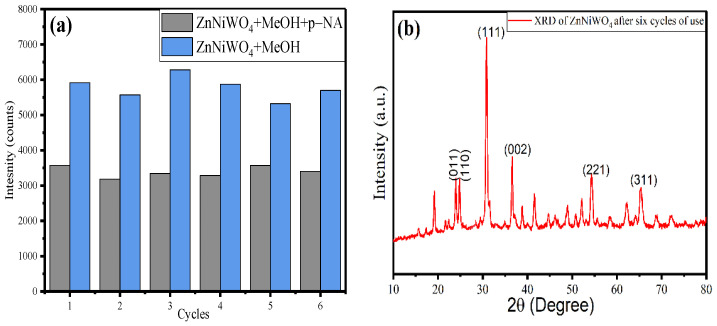
(**a**) Reusability test for ZnNiWO_4_ towards p-NA sensing. (**b**) XRD of ZnNiWO_4_ after six cycles of use.

In order to evaluate the sensing capacity of a sensor, the recyclable usability is an important property of the material. Fluorescence titration experiments were performed for ZnNiWO_4_ towards p-NA (50 μM) in a repeated mode. After cycle 1, the material was washed with methanol three to four times, dried in an oven, and then dispersed in methanol to observe the fluorescence intensity. Then, for cycle 2, the material was again tested for the sensing of p-NA, filtered, and then washed. This procedure was recorded until six cycles of reusability, and the obtained results are given in Figure 13. The black column bar represents the fluorescence intensity of ZnNiWO_4_ with p-NA, while the red column bar represents the fluorescence intensity of ZnNiWO_4_ without the p-NA. It can be seen from the results that, for up to six repeatable cycles of use, there was no appreciable change in the fluorescence intensity of the synthesized material, which suggested that the material was highly stable towards the sensing of p-NA; this was supported by XRD analysis. The XRD spectra of the material after six cycles of use are given in Figure 13b, which shows no change in the structure of the material.

Table 3 compares the LOD values for p-NA-associated sensor materials, by various methods, with the outcomes of present study. It was concluded, based on the data, that the synthesized ZnNiWO_4_ NPs had high sensitivity and LOD values as compared to other methods or materials reported in the literature.

## 4. Conclusions

In the present study, ZnWO_4_, NiWO_4_, and mixed metal ZnNiWO_4_ NPs were synthesized through a hydrothermal process at 180 °C for 12 h. The synthesized nanoparticles were characterized by FTIR, XRD, SEM–EDX mapping, TEM, XPS, and PL spectroscopic techniques. The FTIR results well explained the formation of Zn–O–Ni and W–O types of bonding in mixed metal ZnNiWO_4_ NPs. The XRD results revealed a distorted monoclinic structure of the mixed metal ZnNiWO_4_ NPs with a reduced intensity due to the superposition of Ni in the crystal structure and 13.67 nm as crystallite size at d_111_ peak, which was also supported by TEM analysis. The as-synthesized ZnWO_4_, NiWO_4_, and mixed metal ZnNiWO_4_ NPs exhibited maximum fluorescence emission with methanol as a solvent and were most sensitive towards p-nitroaniline among various nitroaromatic compounds. The order of detection was found to be p-NA > 2-NP > m-NP > 4-NB. With an increase in the concentration of p-NA from 25 μM to 1000 μM, there was a slight decrease in the fluorescence intensity of the synthesized nanoparticles, suggesting an efficient quenching effect. The quenching effects for ZnWO_4_, NiWO_4_, and ZnNiWO_4_ NPs were found to be 93%, 94%, and 98%, respectively. The high value of the Stern–Volmer constant Ksv with regression constant, given in Table 2, for ZnNiWO_4_ (0.018) as compared to ZnWO_4_ (0.015) and NiWO_4_ (0.016) suggested that mixed metal tungstate nanoparticles were proven to be better sensors for p-NA. The limit of detection (LOD) value for p-NA was found to be 1.93 × 10^−8^ M for ZnWO_4_, 2.17 × 10^−8^ M for NiWO_4_, and 2.98 × 10^−8^ M for ZnNiWO_4_, respectively. This work provides a suitable means to develop a new class of potential metal-doped tungstate nanocomposite materials for detecting and sensing various toxic and carcinogenic organic pollutants with high efficiency and cost effectiveness through the combination of experimental and theoretical perspectives.

## Figures and Tables

**Table 1:** XRD parameters of the synthesized ZnWO_4_, NiWO_4_, and ZnNiWO_4_ NPs.

Component	2θ	FWHM (β_hkl_)	Interlayer Spacing (d_111_) (A°)	Size of Crystal (nm) at (111)	Dislocation Density (δ) × 10^19^ Lines (m^−2^)	% Crystallinity (%)
NiWO_4_	31.02	0.52	2.88	15.81	1.21	72.83
ZnWO_4_	30.62	0.57	2.91	14.43	1.18	71.35
ZnNiWO_4_	30.77	0.60	2.90	13.67	1.19	60.87

**Table 2:** Stern–Volmer parameters calculated for ZnWO_4_, NiWO_4_, and ZnNiWO_4_ NPs.

Material	K_SV_	R^2^	LOD (M)
ZnWO_4_	0.015	0.98	1.93 × 10^−8^
NiWO_4_	0.016	0.98	2.17 × 10^−8^
ZnNiWO_4_	0.018	0.97	2.98 × 10^−8^

**Table 3:** Comparison of LOD with the literature.

Sensors	Methods	LOD (M)	Reference
Zn (II)-MOF	Fluorescence	4.7 × 10^−5^	[44]
ZnO NRs/fluorine-doped tin oxide	Electrochemical	0.5 × 10^−6^	[45]
Copper nanoparticles-embedded chitosan	Electrochemical	0.37 × 10^−6^	[46]
1,2,3-triazolyl PTPTB	Fluorescence	4.2 × 10^−6^	[47]
Au-on-Pd NP	Voltametric	0.17 × 10^−6^	[48]
Cucurbituril-modified CdTe quantum dots (CB@QDs)	Fluorescence	6 × 10^−8^	[49]
Chitosan-Ag NPs/CPE	Voltametric	0.86 × 10^−6^	[50]
ZnNiWO_4_ NPs	Fluorescence	2.98 × 10^−8^	Present Study

## Data Availability

Data is contained within the article.
